# Bacterial Biomolecules Drive Extracellular DNA Adsorption onto Ferrihydrite: Interfacial Interactions and Spectroscopic Insights

**DOI:** 10.1021/acs.langmuir.6c00302

**Published:** 2026-06-02

**Authors:** Mateusz Skalny, Jakub Czeremuga, Maciej Roman, Tomasz P. Wróbel, Lukasz Dziewit, Tomasz Bajda

**Affiliations:** † Faculty of Geology, Geophysics and Environmental Protection, 513110 AGH University of Krakow, Mickiewicza 30, 30-059 Krakow, Poland; ‡ SOLARIS National Synchrotron Radiation Centre, 37799 Jagiellonian University, Czerwone Maki 98, 30-392 Krakow, Poland; § Laboratory of Applied Microbial Ecology, Institute of Bioengineering, Faculty of Biology, 49605 University of Warsaw, Miecznikowa 1, 02-096 Warsaw, Poland

## Abstract

The reactivity of extracellular DNA (exDNA) at the mineral interface, modulated by interactions with other biomolecules, may play an important role in governing the fate of genetic information in the environment. However, there is a limited understanding of exDNA-mineral interactions with biomolecule-enriched matrices. Herein, we investigate the mechanisms of exDNA adsorption onto ferrihydrite in the presence of bacterial lysate biomass. The interactions within the system were explored by combining exDNA adsorption studies, zeta potential measurments, spectroscopic studies, and advanced spectrochemical imaging by scattering-type Scanning Near-Field Optical Microscopy (s-SNOM). Bacterial lysate biomass is found to enhance the immobilization efficiency of exDNA by the ferrihydrite. A key role in this process is played by proteins bearing positively charged groups, which facilitate interactions and co-adsorb with exDNA. When the bacterial biomass concentration reaches 14.03 μg/mL, the exDNA removal efficiency nearly doubles relative to the system with sole ferrihydrite. The effect of biomolecules in promoting exDNA adsorption onto ferrihydrite is further demonstrated by s-SNOM. The spectral bands of biomolecules tend to overlap and co-occur in specific regions of conjugates as biomass concentration increases. Our results provide important mechanistic insight into the process potentially influencing the fate of exDNA in environments such as biofilms, soils, and sediments.

## Graphical abstract

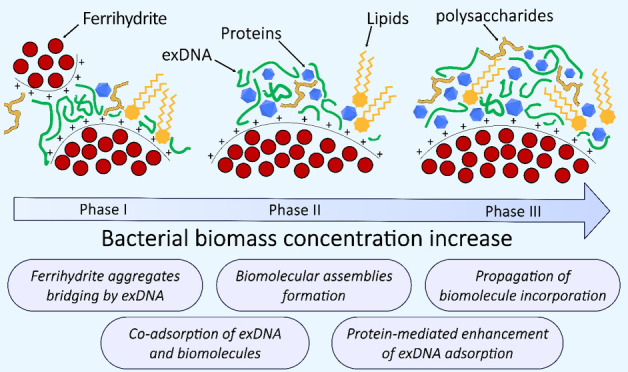

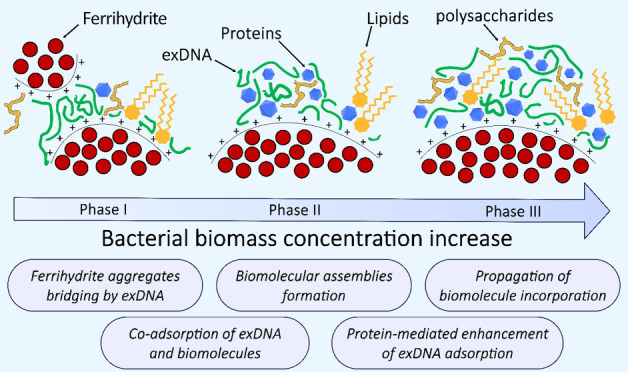

## 1 Introduction

Environmental DNA (eDNA) represents a sum of DNA from all of the compartments of a given environment. In aquatic, soil, and sediment systems, it is a complex mixture of intracellular (iDNA) and extracellular DNA (exDNA).^1,2^ iDNA plays a crucial role in an organism’s functioning, as it is responsible for the storage and transfer of genetic information.^3^ exDNA contributes to horizontal gene transfer (HGT) by serving as a substrate for natural transformation, enabling the uptake of genetic material from the environment. It also serves as a structural agent in biofilms and soils, participates in immune defense, and is even considered a nutrient source for microorganisms.^4,5^ exDNA may be of various origins, i.e., chromosomal or plasmidic, and may be associated with bacterial debris, confined in biofilms, or floating freely in water streams.^1^ Upon release into the environment, exDNA may also interact with mineral surfaces. Processes at the mineral interface have been shown to govern the fate of exDNA by protecting it from degradation,^6,7^ facilitating its transport,^8^ or controlling its uptake by microorganisms.^9^ Especially the last one is an emerging issue because of the potential dissemination of antibiotic resistance genes (ARGs) in the environment. Since exDNA may serve as a vector for ARGs,^10^ and when combined in mineral association, the ARG transfer process may be altered.^11^

The exDNA-mineral interactions depend on both the characteristics of the involved mineral phases and on the conditions of the medium in which adsorption occurs. Parameters such as surface charge, porosity, and the availability of adsorption sites are crucial for the exDNA accumulation potential of minerals.^12–14^ Of equal importance in governing exDNA adsorption capacity and mechanism are pH of the solution, presence of ions, organic molecules, and other biomolecules.^15,16^ Among the wide variety of minerals, iron-based minerals are particularly important, as they exhibit a high affinity for P-bearing molecules, including exDNA. Iron oxides and iron oxy-hydroxides may form strong Fe–O–P bonds with the DNA phosphate backbone, resulting in its selective and efficient accumulation.^17,18^ This may indicate a particular role of Fe phases in governing exDNA fate, accumulation, and reactivity across the ecosystem. While studies evaluated the interfacial reaction of exDNA with goethite, hematite, and lepidocrocite,^17–19^ ferrihydrite remains one of the least studied iron minerals with respect to DNA adsorption. To date, only a single study has compared its reactivity with that of other common minerals.^20^ Due to its unique textural properties, including nanometric crystallite size and high specific surface area, as well as the high availability of surface hydroxyl groups, ferrihydrite may efficiently trap phosphorus-bearing molecules. Moreover, its common occurrence in soils and sediments makes it a critical component potentially governing the fate of exDNA in the environment.^21^

In natural matrices, exDNA is typically a component of organic matter, coexisting with other biomolecules, including proteins, lipids, polysaccharides, and cell wall debris.^22,23^ The interactions between exDNA and these biomolecules are inseparable factors that potentially alter its behavior and reactivity, as well as influence interactions with mineral surfaces^16,24^ . For example, as noted for cellular wall debris, which promoted adsorption and binding of exDNA to the clay mineral surface, resulting in protection from enzymatic degradation.^25^ This may be especially evident in settings such as biofilms, organic-rich sediments, or wastewaters.^26,27^ In such matrices, exDNA may form supramolecular associations with other biomolecules as demonstrated by Schmidt and Martínez, who showed that DNA formed associations with bovine serum albumin through interactions with its positively charged domains and that these influenced adsorption on goethite.^16^ Potentially, exDNA may also form associations with other biomolecules, e.g., polysaccharides or lipids, or be adhered to bacterial cells or cell fragments.^28,29^ This may lead to variations in association characteristics and adsorption mechanisms on mineral surfaces. Such processes may affect the fate of genetic information confined in exDNA and are insufficiently described in scientific literature. Thus, the role of biomolecules in modulating exDNA adsorption onto mineral surfaces remains underexplored. This is particularly important for ferrihydrite, which exhibits strong potential for biomolecule immobilization due to its positive surface charge and the development of textural features.

To address this knowledge gap, our study aims to assess the interactions of exDNA with ferrihydrite in the presence of biomolecules of bacterial origin. This is achieved through a set of adsorption experiments investigating the influence of bacterial lysate at varying concentrations on exDNA adsorption onto ferrihydrite, combined with spectroscopic analysis using Fourier transform infrared spectroscopy (FTIR) and advanced spectral imaging via scattering-type scanning near-field optical microscopy (s-SNOM). The key novelty of the study lies in the determination of potential interactions between biomolecules (proteins, lipids, and polysaccharides) and exDNA during adsorption and ferrihydrite-biomolecule conjugates formation. We determined potential mechanisms of biomolecule assembly on ferrihydrite using ζ-potential measurements and assessed how these mechanisms influence the efficiency of exDNA immobilization.

## 2 Methods and Materials

### 2.1 Materials

In all experiments, as an exDNA source, the sodium salt of salmon sperm DNA (Sigma-Aldrich) was used. The purity of the exDNA solution was assessed spectrophotometrically using a HITACHI U-5100 instrument (Tokyo, Japan) by measuring the 280/260 nm absorbance ratio, which yielded a value exceeding 1.8, indicating proper purity. The exDNA exhibited a chain length >11,000 bp, as determined by gel electrophoresis (Figure S1) using Bio-Rad PowerPac (USA, California). Hydrochloric acid (HCl, 35–38%) and tris­(hydroxymethyl)­aminomethane (Tris Buffer, >99%) were obtained from Chempur, sodium hydroxide (NaOH, >98,8%) and Iron­(III) nitrate nonahydrate (Fe­(NO_3_)_3_·9H_2_O, >99%) were obtained from POCH.

### 2.2 Ferrihydrite Synthesis

Ferrihydrite was synthesized by precipitation using the protocol of Schwertmann and Cornell.^30^ Briefly, 40 g of Fe­(NO_3_)_3_·9H_2_O was dissolved in 500 mL of redistilled water, and 1 M KOH was added dropwise until the pH reached 7. This resulted in the formation of a dark brown precipitate, which was collected by centrifugation, washed several times with redistilled water, and dried in an oven at 50 °C. The material was ground using an agate mortar to a fine powder. The purity of ferrihydrite was confirmed by X-ray diffraction (XRD) and FTIR (Figure S2). The obtained material is a 2-line ferrihydrite with an average crystallite size of 1.105 nm estimated by the Debye–Scherrer equation (eq S1, Supporting Information). Ferrihydrite textural properties were analyzed by N_2_ adsorption/desorption, and its specific surface area was 316.6 m^2^/g (Figure S2 and Table S1). Details on the ferrihydrite analysis are given in the Supporting Information (Methods S2).

### 2.3 Lysed Bacterial Biomass Preparation

For the preparation of lysed bacterial biomass used as a matrix in adsorption experiments, *Escherichia coli* DH5α (Gram-negative), exopolysaccharide-producing *Pseudomonas protegens* ML15 ^31,32^ (Gram-negative), and Gram-positive *Bacillus subtilis* ANT_WA51 ^33^ were chosen. Cultures were grown overnight in 200 mL of Luria–Bertani medium. Then, each culture was centrifuged at 16,639 × *g* for 10 min to obtain a pellet and resuspended in 15 mL of 0.85% w/v NaCl solution. Bacterial suspensions were sonicated on ice to prepare bacterial lysates. The sonication (Sonics Vibra Cell VCX130) was performed at 20 kHz and 35% amplitude. The treatment involved a cycle of 30 s of sonication followed by a 30 s break, repeated for 10 min. This treatment was repeated three times for Gram-negative strains and four times for Gram-positive bacteria. After sonication, all lysates were combined and diluted into a single solution, which will later be referred to as the bacterial biomass. The final concentration of lysed bacterial biomass was 706 μg/mL (calculated from the matrix dry mass).

### 2.4 Adsorption Experiments

All solutions for adsorption experiments used in this study were prepared in 10 mM Tris buffer, made with redistilled water, adjusted to pH 6.5, and filtered through 0.22 μm filters. The use of a buffer ensured proper exDNA solubility and maintained a constant pH after the addition of bacterial biomass and throughout the adsorption process. Prior to adsorption experiments, a 25 μg/mL exDNA stock solution and a 25 mg/mL ferrihydrite dispersion were prepared. Before each use, ferrihydrite dispersion was mobilized by 15 min of sonication and 30 min of stirring on a magnetic stirrer. All experiments used an initial exDNA concentration of 0.5 μg/mL to achieve adequate detection by fluorometry and to ensure relevance to environmental concentrations.^34^ All adsorption experiments were conducted in parallel triplicate to ensure replicability, and averages with standard deviations were calculated. In all experiments, control samples containing only exDNA solution were also prepared and showed no change in exDNA concentration. The exDNA adsorption capacity—*Q*_e_ (mg/g) and removal efficiency—*E* (%) were calculated using formulas [Disp-formula eq1] and 2, respectively.

$$Q_{e}=\frac{(C_{0}-C_{e})\times V}{M}$$ 1

$$E=\frac{\left(C_{0}-C_{e}\right)}{C_{0}}\times 100$$ 2

where *C*_0_ (mg/L) is the starting exDNA concentration, *C*_e_ (mg/L) is the equilibrium exDNA concentration, *V* (L) is the volume of the used solution, and *M* (g) stands for the mass of the adsorbent.

#### 2.4.1 Ferrihydrite Dosage Influence on exDNA Adsorption and Adsorption Isotherm

The influence of ferrihydrite dosage on exDNA adsorption efficiency was investigated in batch experiments by pipetting a proper volume of ferrihydrite dispersion to a 0.5 μg/mL exDNA solution to achieve final ferrihydrite concentrations of 0.025, 0.050, 0.075, 0.1, 0.15, 0.2, 0.3, and 0.5 mg/mL. Tubes were shaken at 250 rpm for 60 min on a laboratory shaker, then centrifuged at 4500 rpm for 5 min, and the supernatant was sampled for exDNA concentration analysis. exDNA concentrations in solutions after adsorption were determined using a Qubit fluorometer 4 (Thermo Fisher) using 1× dsDNA high-sensitivity assay. Briefly, 10 μL of solution was pipetted to 190 μL of high-sensitivity buffer in Qubit assay tubes and analyzed on a precalibrated Qubit 4 fluorometer.

The adsorption isotherm was constructed by preparing 5 mL of exDNA solutions at concentrations of 0.5–10 μg/mL in tubes, then adding ferrihydrite dispersion to obtain its final concentration of 0.2 mg/mL. Tubes were shaken at 250 rpm for 60 min on a laboratory shaker, then centrifuged at 4500 rpm for 5 min, and the supernatant was sampled for exDNA concentration analysis as described above. The experimental results were modeled using Langmuir (eq S2) and the Freundlich (eq S3) equations.

#### 2.4.2 Bacterial Biomass Influence on exDNA Adsorption onto Ferrihydrite

The influence of bacterial biomass on exDNA adsorption onto ferrihydrite was also investigated by a batch experiment. Briefly, ferrihydrite dispersion and bacterial biomass solution were simultaneously pipetted into tubes containing 0.5 μg/mL exDNA solution to achieve 0.05 mg/mL of ferrihydrite and 0, 0.31, 0.46, 0.69, 1.04, 1.56, 2.34, 3.51, 7.02, 10.52, 14.03 μg/mL of bacterial biomass. Tubes were shaken at 250 rpm for 60 min on a laboratory shaker, then centrifuged at 4500 rpm for 5 min, and the supernatant was sampled for exDNA concentration analysis. In parallel, control experiments were conducted using the same protocol without the addition of ferrihydrite. The exDNA concentration was analyzed in supernatants as described above. The DNA content in the bacterial biomass solution added to the final system was marginal and did not affect the final exDNA concentration.

#### 2.4.3 exDNA Adsorption Kinetics onto Ferrihydritein the Presence of Bacterial Biomass

exDNA adsorption kinetics with and without a bacterial biomass matrix were investigated by the simultaneous addition of ferrihydrite dispersion and bacterial biomass into the containers with a 0.5 μg/mL exDNA solution. The final ferrihydrite concentration was 0.1 mg/mL, and bacterial biomass concentrations were 0, 1.04, and 3.51 μg/mL. Containers were shaken at 250 rpm on a laboratory shaker, and solutions were sampled by syringes and filtered through 0.45 μm syringe filters at 0.5, 1, 3, 5, 8, 15, 30, 45, 60, 90, 120, 180, and 240 min of the experiment. Simultaneously, control experiments were conducted using the same protocol without the addition of ferrihydrite. Filtering the exDNA solution through 0.45 μm syringe filters has been shown not to affect exDNA concentration. The exDNA concentration was analyzed in supernatants as described above. The adsorption kinetic data were fitted using pseudo-first-order (eq S4) and pseudo-second-order (eq S5) kinetic models. Detailed modeling descriptions are provided in the Supporting Information (Methods S3).

#### 2.4.4 The pH Influence on exDNA Adsorption onto Ferrihydrite in the Presence of Bacterial Biomass

The influence of pH on exDNA adsorption, with and without bacterial biomass, was investigated by first preparing tubes with 0.5 μg/mL exDNA solutions at pH 5, 6, 7, 8, and 9 in 10 mM Tris buffer. Subsequently, ferrihydrite dispersion and bacterial biomass were simultaneously pipetted into tubes containing exDNA solutions at varying pH. The addition of ferrihydrite and bacterial biomass did not influence the initial pH of the exDNA solution. The final ferrihydrite concentration was 0.1 mg/mL, and the final bacterial biomass concentrations were 0, 1.04, and 3.51 μg/mL. Tubes were shaken for 60 min at 250 rpm on a laboratory shaker, then centrifuged at 4500 rpm for 5 min, and the supernatant was sampled for exDNA concentration analysis. The exDNA concentration was then measured in the supernatants as described above.

#### 2.4.5 Sedimentation Experiments

The sedimentation rates of ferrihydrite were investigated using the turbidimetric method.^35^ The ferrihydrite suspension, exDNA solution, and bacterial biomass were pipetted into a spectrophotometric cuvette, shaken by hand for 5 s, and then inserted into the spectrophotometer (HITACHI U-5100). Then, turbidity was recorded at 600 nm every 5 min over 60 min, according to the method proposed by Szewczuk-Karpisz et al.^36^ The concentration of ferrihydrite in the final solution was 0.05 mg/mL, the exDNA concentration was set to 0.5 μg/mL, and bacterial biomass concentrations were 0, 1.04, 3.51, and 14.03 μg/mL.

### 2.5 Zeta Potential Measurements

After adsorption at varying ferrihydrite dosages and bacterial biomass concentrations, the centrifuged materials were washed with 5 mL of 10 mM Tris buffer, resuspended in 5 mL of 10 mM Tris buffer, and vortexed for 30 s. The resulting suspension was pipetted into sample cells and measured for zeta potential using a Zetasizer Nano ZS (Malvern, Worcestershire, United Kingdom).^37^ Additionally, the raw bacterial biomass at varying concentrations and the 0.5 μg/mL exDNA solution were measured for zeta potential. All measured zeta potential distributions were unimodal, indicating a uniform surface charge distribution among the particles in the analyzed samples. The zeta potential was calculated using the Smoluchowski formula.^37^

### 2.6 Characterization of Solid Samples after Adsorption

To investigate the exDNA adsorption mechanism onto ferrihydrite in the presence of bacterial biomass, materials after adsorption at varying bacterial biomass concentrations were analyzed using FTIR, Transmission Electron Microscopy (TEM), and s-SNOM. The following samples were used for each type of analysis: (i) raw ferrihydrite (Fr), (ii) ferrihydrite after exDNA adsorption (Fr-DNA), (iii) ferrihydrite after exDNA adsorption in bacterial biomass medium with a concentration of 1.04 μg/mL (Fr-DNA-ML), (iv) ferrihydrite after exDNA adsorption in bacterial biomass medium with a concentration of 3.51 μg/mL (Fr-DNA-SL), (v) ferrihydrite after exDNA adsorption in bacterial biomass medium with a concentration of 14.03 μg/mL (Fr-DNA-DL). ML, SL, and DL designations originate from the internal sample code and correspond to systems with bacterial biomass concentrations of 1.04, 3.51, and 14.03 μg/mL, respectively. This selection of samples allowed for comparison of ferrihydrite exposed only to exDNA and ferrihydrite after exDNA adsorption under the influence of varying bacterial biomass concentrations. Before each analysis, samples were washed with deionized water to prevent buffer crystallization.

The FTIR spectra of the listed samples were collected using a Nicolet 6700 spectrometer (Thermo Fisher, Waltham, MA, USA) in the 4000–400 cm^–1^ range at a resolution of 4 cm^–1^. Before analysis, the samples were dried at 35 °C, and KBr pellets were prepared by homogenizing the sample with KBr powder in an agate mortar.

TEM observations were performed using a Tecnai TF20 X-TWIN (FEG) microscope (FEI, Hillsboro, OR, USA) at an accelerating voltage of 200 kV. Prior to analysis, samples in the form of deionized water dispersion were drop-cast onto carbon-coated copper TEM grids and dried in air at room temperature.

AFM and s-SNOM images were recorded using a neaSCOPE microscope (attocube systems AG, Germany). The microscope is equipped with a Quantum-Cascade Laser (QCL) with a tuning range of 1725–925 cm^–1^ for continuous emission, an asymmetric Michelson interferometer, a high-NA (0.46) parabolic mirror, and an MCT liquid-nitrogen-cooled IR detector. ARROW-NCPt (285 kHz, NanoWorld, Switzerland) monolithic silicon AFM tips with a platinum/iridium coating and an apex radius of approximately 25 nm were used for sample imaging. For each wavenumber, the laser power was set to 1 mW. Pseudoheterodyne (Ps-Het) detection mode was used to collect optical amplitude and phase signals. Optical signals were acquired for the demodulated second harmonics of the AFM tip frequency. The optical phase signal of the sample was referenced to the Si substrate using the formula φ_sample_–φ_Si_. Prior to analysis, the silicon wafers used as substrates were cleaned with a 3:1 mixture of H_2_SO_4_ and H_2_O_2_ and rinsed with deionized water. Subsequently, samples were pipetted onto silicon wafers, dried in air at room temperature, and analyzed by s-SNOM.

## 3 Results and Discussion

### 3.1 exDNA Adsorption on Ferrihydrite

First, the effect of ferrihydrite dosage on exDNA adsorption was assessed (Figure 1). At the lowest applied ferrihydrite concentration (0.025 mg/mL), exDNA is removed with ∼25% efficiency, and the adsorption capacity reaches 5.36 μg/mg. With increasing ferrihydrite dosage, the exDNA removal efficiency reaches ∼87.5% and 100% at 0.1 and 0.15 mg/mL, respectively. The range of determined adsorption capacity values aligns with the outcomes of Lu et al., who applied nano zerovalent iron for DNA adsorption at similar adsorbent dosage and DNA concentrations,^38^ thereby confirming the similar immobilization capacity of Fe-based nanomaterials toward DNA molecules. The exDNA adsorption isotherm on ferrihydrite (Figure S3) shows relatively high linearity over a wide range of exDNA concentrations and yields a better fit to the Freundlich model than to the Langmuir model (Table S2). This may indicate that, with an increasing amount of adsorbed exDNA on the ferrihydrite surface, new active sites are formed, leading to the formation of an adsorbate multilayer.^39,40^

**Figure 1:**
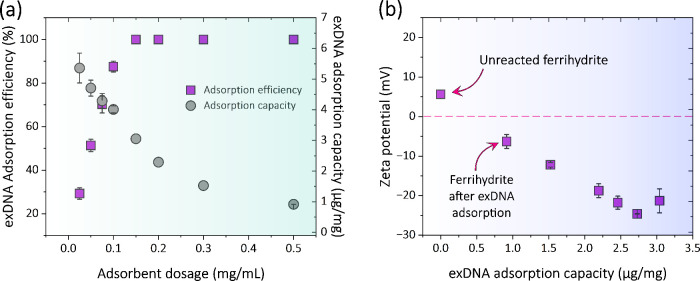
Ferrihydrite dosage effect on the exDNA adsorption capacity and efficiency (exDNA *C*_0_: 0.5 μg/mL, reaction time: 60 min) (a), zeta potential of ferrihydrite particles after exDNA adsorption (b). Error bars represent the standard deviation of independent samples.

Under applied experimental conditions, the ferrihydrite surface is positively charged,^41^ whereas exDNA exhibits a net negative charge,^42^ which is grouped in the exDNA backbone.^43^ This results in strong electrostatic attraction, leading to charge neutralization during adsorption. The interactions in such a system can be well assessed using zeta potential measurements, which can yield insights into the underlying adsorption mechanism. The zeta potential of raw ferrihydrite in buffer matrix at a pH of 6.5 is positive (∼8 mV) and drops to ∼−5 mV at the lowest studied exDNA loading (1.6 μg/mg) (Figure 1b). exDNA adsorption on the ferrihydrite aggregates may extend their interfacial layer and shift the slipping plane away from the surface, lowering the absolute value of the zeta potential. Therefore, a reduction in the electrokinetic potential of the formed conjugates after adsorption compared to raw ferrihydrite confirms the immobilization of exDNA molecules on its surface.^41^

The reaction of exDNA or nucleotides with iron oxyhydroxides occurs via chemisorption.^17,44^ In this process, ligand exchange occurs between the exDNA phosphate backbone and the protonated hydroxyl groups of ferrihydrite, leading to the formation of Fe–O–P bonds.^17,18,45^ With increasing exDNA loading on the ferrihydrite surface, the ζ-potential further decreases, and this seems to be nearly linear in the analyzed loading range. A gradual decrease in the particle’s ζ-potential indicates that exDNA incorporation is proceeding despite the intensification of repulsion forces.^14^ Such a trend in electrical potential at the slipping plane of ferrihydrite aggregates may suggest that exDNA does not react along its entire length with the surface or that, to some extent, its immobilization occurs via intramolecular interactions between exDNA molecules and negatively charged phosphate groups are not fully neutralized by the ferrihydrite surface. Thus, the exDNA multilayer is likely to form on the ferrihydrite surface, as previously indicated by a better fit to the Freundlich model than to the Langmuir model.

Changes in the charge of particles and the presence of polyelectrolyte molecules (exDNA) in a colloidal system affect the stability of the dispersion.^46^ The reaction of ferrihydrite with exDNA results in faster settlement of particles (Figure S4—Fr and Fr-DNA), which is due to the formation of conjugates, consisting of multiple ferrihydrite aggregates bonded by exDNA (Figure 1b). The conjugate formation process is driven by exDNA’s polyelectrolyte nature,^47^ in which it acts as a particle bridging agent. As shown by TEM images, ferrihydrite aggregates form larger nebulous-like aggregates after exposure to exDNA (Figure S5b).

### 3.2 Role of Bacterial Biomass in Facilitating exDNA Adsorption on Ferrihydrite

In our experimental setup, which investigates bacterial lysate as an agent influencing exDNA adsorption onto ferrihydrite, various biomolecules may interact with exDNA, thereby forming supramolecular associations. As shown by Schmidt and Martínez, DNA formed such associations with bovine serum albumin via interaction with its positively charged fragments.^16^ In contrast to this study, in our setup, supramolecular associations may comprise not only exDNA and proteins but also polysaccharides, lipids, or even larger cellular compartments.^48^

Figure 2a shows the effect of bacterial biomass on the removal efficiency of exDNA by ferrihydrite. Increasing bacterial biomass concentration enhances exDNA removal in the ferrihydrite system across the entire examined range. Removal efficiency increases gradually, and at the maximal tested bacterial biomass load (14.03 μg/mL), it reaches 89% (8.7 μg/mg), which is nearly double that of the system containing only ferrihydrite, showing 46% exDNA removal efficiency and a capacity of 4.5 μg/mg (Figure S6). Furthermore, it is essential to note that, in a system without ferrihydrite, exDNA is also removed when the bacterial biomass concentration exceeds ∼0.69 μg/mL. At relatively low bacterial biomass concentrations, a significant difference in exDNA removal efficiency between the system with and without ferrihydrite is observed. While at bacterial biomass concentrations ≥2.34 μg/mL, both systems exhibit similar exDNA incorporation. This suggests that exDNA may be efficiently adsorbed by biomolecular associations or adhere to larger cell fragments, even in the absence of ferrihydrite particles. The ionic strength, which is also a driver of exDNA adsorption on mineral surfaces,^46,49^ increases only slightly with the addition of bacterial biomass in the range of 1.23–1.28 mS/cm (Figure S7). This indicates that direct intermolecular interactions between bacterial biomass and exDNA are key factors in increasing exDNA incorporation into conjugates formed in the system, rather than aggregation driven by an increase in the ionic strength of the reaction solution.

**Figure 2:**
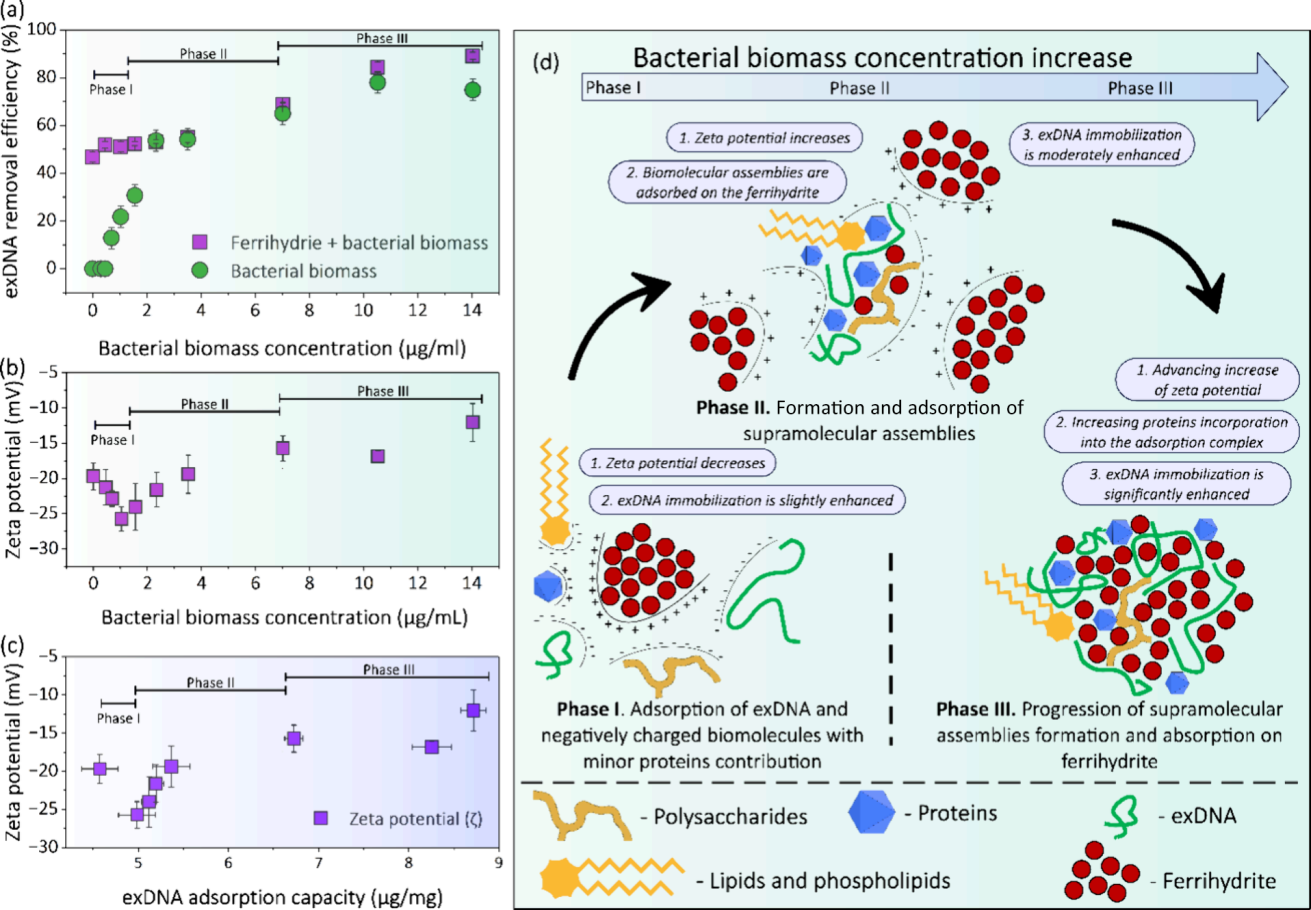
Influence of bacterial biomass concentration on the adsorption of exDNA onto ferrihydrite (exDNA *C*_0_: 0.5 μg/mL, reaction time: 60 min, ferrihydrite concentration: 0.05 mg/mL) (a), zeta potential of ferrihydrite particles after exDNA adsorption under increasing bacterial biomass concentration (b), zeta potential of ferrihydrite particles with increasing exDNA loading (caused by bacterial biomass) (c), the visualization of potential ferrihydrite, exDNA, and biomolecules interactions in the system under increasing bacterial biomass concentration (d). Error bars represent the standard deviation of independent samples.

Figure 2b,c shows the zeta potential of ferrihydrite-exDNA conjugates formed under increasing concentrations of bacterial biomass and zeta potential correlation with exDNA adsorption capacity onto ferrihydrite, respectively. An interesting trend was observed: initially, the zeta potential of the conjugates decreased with increasing bacterial biomass concentration up to 1.04 μg/mL. As biomass concentration increases further, zeta potential starts to rise and continues to increase to the highest biomass loading. This shows a significant impact of biomolecules contained in bacterial biomass on the electrostatic potential at the slipping plane of ferrihydrite-biomolecule conjugates and its relationship with the exDNA adsorption capacity. An increase in zeta potential observed during enhanced exDNA immobilization onto ferrihydrite by bacterial biomass indicates the co-adsorption of positively charged molecules from the lysate matrix, most likely proteins. The raw exDNA and bacterial biomass both exhibit a negative net zeta potential. Specifically, exDNA shows a value of approximately −32.7 mV, while bacterial biomass ranges from −2.55 to −7.45 mV, depending on its concentration (Figure S8). Therefore, an increase in zeta potential of Ferrihydrite-exDNA conjugates formed in bacterial biomass indicates a selective, to a certain extent, uptake of proteins from the biomass matrix in relation to other biomolecules.

Further insights into the colloidal properties of the system are provided by sedimentation experiments assessing the colloidal stability of ferrihydrite exposed to exDNA and bacterial biomass (Figure S4). After exDNA adsorption onto ferrihydrite in a matrix with a relatively low and moderate bacterial biomass concentration (1.04 μg/mL—Fr-DNA-ML and 3.51 μg/mL—Fe-DNA-SL), the sedimentation rate is slightly slower compared to the Fr-DNA sample. Usually, when the zeta potential of particles approaches zero, a decrease in colloidal stability is observed.^50^ Thus, sedimentation kinetics results are consistent with zeta potential measurements, as the zeta potentials of particles in the Fr-DNA-ML and Fr-DNA-SL samples are, respectively, lower and relatively equal compared to the Fr-DNA sample. Thus, the colloidal stability of ferrihydrite particles after exDNA adsorption at low and moderate bacterial biomass loadings is greater than in the system without bacterial biomass. This can also be explained by the specific colloid-stabilizing properties of the polysaccharides present in bacterial biomass.^51^ Polysaccharides may form a layer-like structure around ferrihydrite particles and aggregates, thereby inducing a steric barrier and promoting colloidal stability. A similar effect was demonstrated for nano ceria particles and EPS rich in polysaccharides.^52,53^ Moreover, the increasing protein content at the particle interface may enhance system stability, as shown by Sheng et al., a sufficiently thick protein layer adsorbed on the surface of hematite nanoparticles stabilizes their dispersion due to steric repulsion.^54^ As the bacterial biomass concentration increases further in the system, sedimentation accelerates significantly for the Fr-DNA-DL sample, indicating markedly lower colloidal stability. This appears to occur despite potential co-adsorption of polysaccharides and proteins and is attributed to a significant increase in zeta potential (Figure 2b) and, potentially, to the growth of conjugate size in the system. Therefore, the obtained results indicate that the bacterial biomass concentration may regulate the colloidal stability of the ferrihydrite–exDNA system.

This combined investigation enabled us to distinguish three phases in the exDNA adsorption process on ferrihydrite, triggered by increasing bacterial biomass load (Figure 2d). In Phase I, characterized by low bacterial biomass concentration, the positively charged surface of ferrihydrite directly reacts with exDNA and potentially with other biomolecules, including lipids, polysaccharides, and proteins. This leads to charge neutralization of the ferrihydrite surface and a decrease in the overall zeta potential of particles in the system. In Phase I, the protein concentration in the system is too low to induce a significant increase in zeta potential or facilitate further adsorption of exDNA and other anionic biomolecules. Then, as the bacterial biomass concentration increases, Phase II begins, during which supramolecular association formation and exDNA immobilization in ferrihydrite conjugates intensify. Various intramolecular interactions may drive these processes. Proteins may interact with exDNA through electrostatic interactions, such as direct attraction to their positively charged moieties,^55,56^ as well as via hydrogen bonding.^57^ To lipids and polysaccharides, exDNA may bind via ion-mediated, hydrophobic, and hydrogen-bonding interactions.^58–60^ Moreover, it is essential to note that cations such as Na^+^, K^+^, Ca^2+^, or Mg^2+^ are present in bacterial biomass^61^ and may complex the exDNA phosphate backbone, shielding its charge and compacting its structure, leading to improved attachment to mineral surfaces.^12,14,62^ As indicated by zeta potential measurements, in Phase II, the net charge of conjugates in the system increases, confirming additional neutralization of exDNA negative charge, e.g., through the enhanced incorporation of proteins into conjugates. Despite charge neutralization, these complexes still adhere to the positively charged ferrihydrite surface, as sedimentation of ferrihydrite particles in the system is influenced.

As the bacterial biomass concentration continues to increase, Phase III begins, during which the growth of supramolecular associations propagates. As shown by TEM images (Figure S5e), conjugates formed at high concentrations of bacterial biomass (Fr-DNA-DL) exhibit net-like structures, in which mineral aggregates are coated and bridged by bacterial biomass. This is due to the incorporation of a substantial amount of biomolecules into the conjugates, thereby resulting in the formation of numerous positively charged adsorption sites (by proteins), which enhance exDNA uptake, particularly in samples obtained from the systems containing ≥7.02 μg/mL of bacterial biomass. Under applied experimental conditions, raw bacterial biomass exhibits a net negative charge (Figure S8). However, proteins in such a matrix may exhibit a wide range of functional groups with varying charges and isoelectric points (pI). For example, cytoplasmic proteins typically have a pI of ∼5–6, whereas membrane proteins may exhibit a pI of ∼8.1.^63^ Despite studies indicating that acidic proteins with pI between 5 and 7 may dominate in bacteria such as *E. coli*,^64,65^ a multimodal distribution of protein pI in cells is usually noted.^66^ Thus, in the bacterial biomass matrix, proteins with differing charge coexist. exDNA in such a system may directly interact with positively charged proteins through electrostatic interactions, and as indicated by Schmidt and Martínez, it may also interact with positively charged moieties of proteins exhibiting net negative charge.^16^

The adsorption kinetics of exDNA in the presence and absence of bacterial biomass is shown in Figure 3. The removal efficiency of exDNA by ferrihydrite increased rapidly to 85% within the first 45 min and gradually reached 90% after 250 min. A similar two-step pattern in the exDNA absorption was previously observed for ferrihydrite.^20^ Such adsorption kinetics may result from the nature of interfacial interactions between exDNA and ferrihydrite aggregates, reflecting the polyelectrolyte nature of the exDNA. In the first adsorption step, exDNA molecules diffuse from the bulk solution to the mineral interface, which is driven by a concentration gradient. In the second, slower adsorption step, exDNA is reorganized at the ferrihydrite surface, and its unbound functional groups are attached to free binding sites.^67^ Moreover, in the second step, the unbound exDNA present in solution may bind to adsorbed exDNA molecules via intramolecular interactions, thereby forming an adsorbate multilayer.

**Figure 3:**
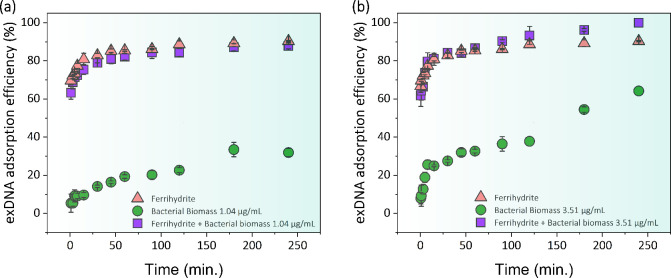
exDNA adsorption kinetics onto ferrihydrite with and without bacterial biomass and onto bacterial biomass alone at concentrations of 1.04 μg/mL (a) and 3.51 μg/mL (b) (exDNA *C*_0_: 0.5 μg/mL, ferrihydrite concentration: 0.1 mg/mL). Error bars represent the standard deviation of independent samples.

At a bacterial biomass concentration of 1.04 μg/mL, the exDNA adsorption kinetics onto ferrihydrite exhibit similar results to those without biomass (Figure 3a). While at a higher bacterial biomass concentration (3.51 μg/mL), greater exDNA removal efficiency is achieved, and in both cases, two-step kinetics is observed (Figures 3b and S9). The adsorption kinetics modeling indicates higher *R* ^2^ values for the pseudo-second-order model compared to the pseudo-first-order model, and that the *k*_1_ and *k*_2_ constants decrease with increasing bacterial biomass concentration in the reaction matrix (Table S3). This is especially evident in the pseudo-second model, which yields *k*_2_ values with with slight variability, as indicated by their standard deviations across samples, while the pseudo-first-kinetic model gave similar *k*_1_ values with coinciding standard deviations. Thus, kinetic modeling indicates a slightly lower exDNA adsorption rate on ferrihydrite in the presence of bacterial biomass, particularly when a significant amount of supramolecular associations form (at a bacterial biomass concentration of 3.51 μg/mL). The lower adsorption rate in such a system may arise from intensified intramolecular interactions at the mineral interface and an increased amount of exDNA incorporated into the adsorbate multilayer due to co-adsorbed proteins. The removal of exDNA by bacterial biomass alone is observed in the kinetic experiment and also exhibits a two-step character. Rapid exDNA removal by bacterial biomass occurs within the first 15 min of the experiment, then the removal proceeds at a slower rate. This confirms the immobilization of exDNA within supramolecular associations and the importance of diffusion processes during biomolecules interactions. However, the presence of ferrihydrite clearly facilitates exDNA immobilization within the bacterial biomass matrix, as its reaction with exDNA proceeds at a higher rate than with bacterial biomass alone.

The adsorption efficiency of exDNA onto ferrihydrite strongly depends on the pH of the reaction solution. As shown in Figure S10, exDNA removal efficiency decreases with increasing pH. A similar trend is typically observed for iron oxides and related minerals when the adsorption of anionic species is assessed.^68,69^ Additionally, non-iron oxide minerals, such as allophanes, show a similar tendency regarding exDNA adsorption.^70^ In the case of ferrihydrite, pH determines the amount of hydroxyl surface species available as active centers for anionic molecules adsorption.^71^ Lower pH results in a greater abundance of protonated hydroxyl groups at the ferrihydrite surface and an overall higher surface charge (Figure S2e), which explains the greater affinity towards exDNA.^72^ pH also regulates the charge of particular biomolecules, such as proteins, and their affinity for surfaces.^73^ The experimental data indicate a slight decrease in exDNA removal efficiency in the presence of bacterial biomass compared to a system containing only ferrihydrite at pH 5 and 6, which is the opposite of that at neutral pH. This indicates that at pH below 6.5, bonding of exDNA directly to the ferrihydrite surface becomes more pronounced relative to co-adsorption in assemblies with biomolecules. Moreover, at slightly acidic pH, biomolecules in the system may compete with exDNA for the active sites of ferrihydrite (protonated hydroxyl groups). At pH values of 6.5, 7, and 8, the enhancement of exDNA immobilization by biomolecules is evident. While, at pH 9, the removal efficiency of exDNA in all systems is substantially low, possibly due to the limited availability of ferrihydrite hydroxyl species. The high dependence of exDNA immobilization within ferrihydrite conjugates on the pH of the reaction solution indicates that the protonated hydroxyl groups of ferrihydrite serve as important adsorption centers for exDNA, whether exDNA is confined within supramolecular complexes or floats freely in solution. Importantly, pH may also regulate the co-adsorption of lipids, polysaccharides, and proteins onto ferrihydrite (due to the presence of negatively charged functional groups), as well as intramolecular interactions between biomolecules in the system (e.g., hydrogen bonding) and the conformation of exDNA.^74^ Therefore, it can influence the bonding of exDNA and biomolecules in the adsorbate multilayer.

### 3.3 Spectroscopic Investigation of exDNA Adsorption onto Ferrihydrite Facilitated by Bacterial Biomass

#### 3.3.1 FTIR Analysis

FTIR was used to investigate changes in the chemical composition of ferrihydrite resulting from the adsorption of exDNA and biomolecules. Figure 4 shows the normalized FTIR spectra of ferrihydrite before and after exDNA adsorption in the presence and absence of bacterial biomass (Fr, Fr-DNA, Fr-DNA-ML, Fr-DNA-SL, and Fr-DNA-DL), ferrihydrite exposed to bacterial biomass at a concentration of 14.03 μg/mL with no exDNA addition (Fr-DL), as well as raw exDNA and bacterial biomass.

**Figure 4:**
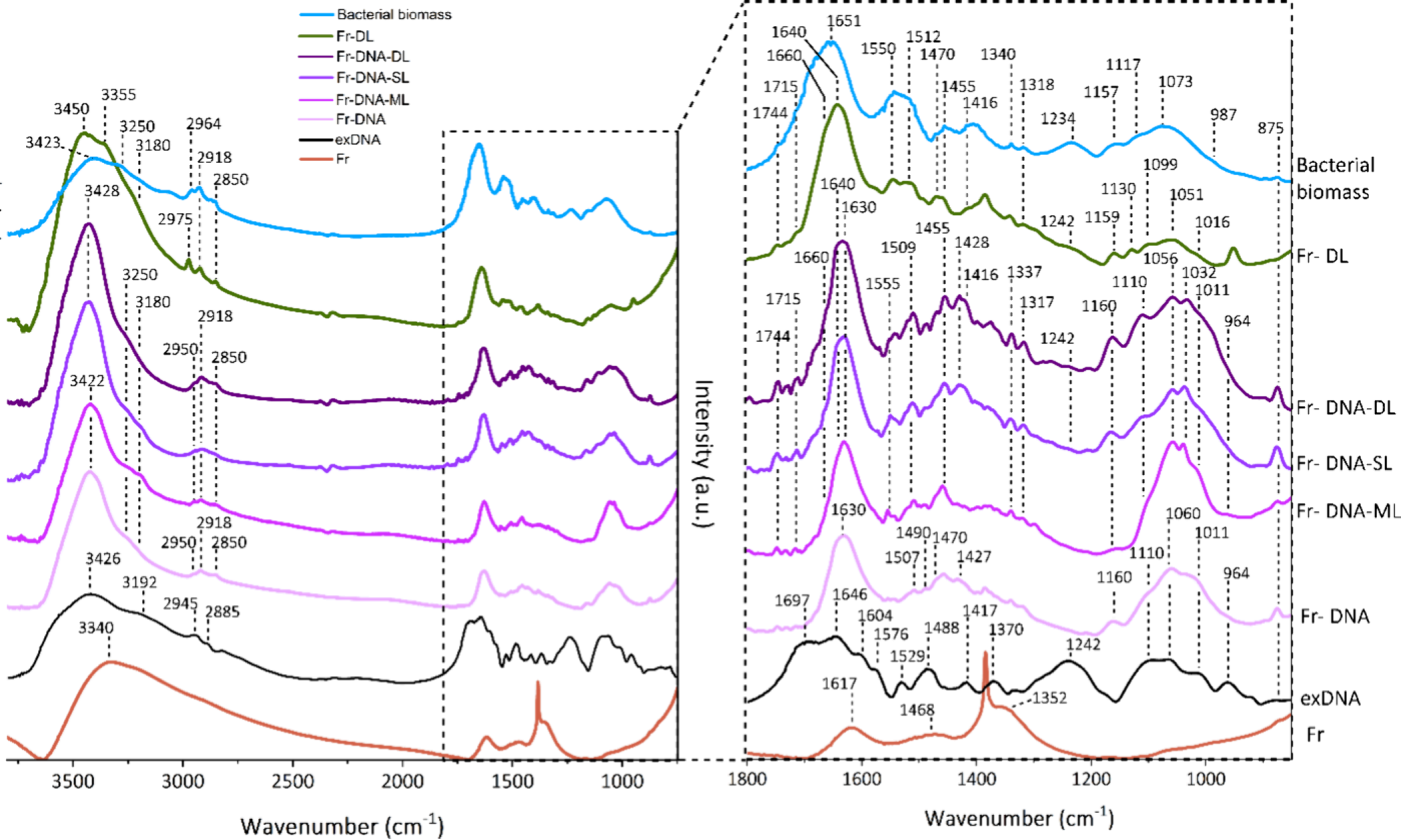
FTIR spectra of Fr, Fr-DNA, Fr-DNA-ML, Fr-DNA-SL, and Fr-DNA-DL samples in 750–3800 and 820–1710 cm^–1^ ranges, with a spectral resolution of 4 cm^–1^. (Additionally shown is sample Fr-DL, which represents ferrihydrite reacted with bacterial biomass at a concentration of 14.03 μg/mL with no exDNA addition and spectra for raw exDNA and bacterial biomass).

Upon exDNA adsorption, new bands appear in the spectra of the ferrihydrite (Fr-DNA). This includes the bands in the 950–1220 cm^–1^ region attributed to C–C stretching vibrations of the deoxyribose (DNA backbone) (964 cm^–1^), Fe–O–P or Furanose vibrations (1011 cm^–1^), C–O/C-C stretching vibrations, or symmetric stretching vibrations of PO_2_^–^ (1060–1110 cm^–1^).^17,74,75^ Then, low-intensity bands in the 1350–1550 cm^–1^ region appear, indicating vibrations of the sugar moieties and nitrogenous base rings, along with base–sugar modes.^18^ A band at 1560–1710 cm^–1^, with a peak at 1630 cm^–1^, is also observed, indicating the presence of adsorbed water. This band potentially overlaps with C=O stretching vibrations of base pairs as well as C=N and C=C vibrations of nucleobase rings.^76^ The determined bands confirm the presence of exDNA immobilized on the ferrihydrite surface.

Comparing the spectra of raw exDNA with those of exDNA adsorbed onto ferrihydrite allows investigation of the exDNA bonding mechanism to the ferrihydrite surface (Figure 4 - exDNA and Fr-exDNA). Several notable band shifts are determined. First, the phosphate-related band at 1060 cm^–1^ increases in intensity relative to the shoulder band at 1110 cm^–1^, upon adsorption. Moreover, the increase in intensity of the band indicative of Fe–O–P (1011 cm^–1^) occurs in relation to neighboring bands. This indicates the adsorption of exDNA via the complexation of phosphate groups located in the exDNA backbone with the Fe active sites of ferrihydrite.^17,77^ Interestingly, the exDNA signal at 1240 cm^–1^, which corresponds to the asymmetric stretching PO_2_^–^ vibration mode, disappears. Such a result was also confirmed by the studies of Huang et al. and Sheng et al., who reported similar phenomena in DNA adsorption on nanometric allophane particles and montmorillonite, respectively.^47,78^ A significant decrease in the intensity of the 1240 cm^–1^ band may indicate strong phosphate complexation and a crucial role for the protonated hydroxyl groups of ferrihydrite in bonding the exDNA backbone. The next significant change after adsorption is observed in the exDNA spectral region from 1400 to 1510 cm^–1^, related to nucleobases. Specifically, the raw exDNA band at 1488 cm^–1^ appears to split into three neighboring bands at 1470, 1490, and 1507 cm^–1^, and the raw exDNA band at 1417 cm^–1^ shifts toward 1427 cm^–1^. This suggests that nitrogenous bases may be potentially involved in the bonding of exDNA molecules to the ferrihydrite surface. For example, nucleobase functional groups (−NH_2_, C=O, C=N) may react with ferrihydrite surface hydroxyl species via hydrogen bonds^79–81^ or interact with its surface via van der Waals forces.^81^ Moreover, potential nucleobase interactions with the ferrihydrite surface are indicated by the band at 1697 cm^–1^, which seems to shift toward lower wavelengths upon exDNA adsorption. However, determining the specific changes in the 1570–1680 cm^–1^ region is difficult due to overlap with absorbed water.

While the binding of exDNA to the ferrihydrite surface via its phosphate backbone appears to be a primary adsorption mechanism,^17^ the nucleobases also seem to react with ferrihydrite hydroxyl species. This dual adsorption mechanism was determined in previous studies for DNA adsorption on the α-Fe_2_O_3_,^18^ SiO*_x_*,^82^ or TiO_2_.^83^ Such interactions are potentially favored for ferrihydrite, which forms aggregates in aqueous solutions. Compared to mineral particles with rigid surfaces and significant crystalline size, such as goethite or TiO_2_,^17,38^ ferrihydrite aggregates are composed of nanometric particles (∼1.1 nm), as shown in Figure S5, and by the Debye–Scherrer equation, and may exhibit varying morphologies, that may change during the adsorption process and adopt new shapes. Thereby, the attachment of polyelectrolytic exDNA molecules may occur to a greater extent on ferrihydrite aggregates compared to minerals with larger particle diameters.^14^

Ferrihydrite, after adsorption in a bacterial biomass matrix (Fr-DNA-ML, Fr-DNA-SL, and Fr-DNA-DL), shows a significant increase in intensity of specific bands in the 950–1200 and 1290–1750 cm^–1^ regions of the spectra (Figure 4). The exDNA characteristic phosphate and furanose-related bands are present. Nevertheless, with increasing bacterial biomass load, the intensities of the shoulder bands at 1032 and 1110 cm^–1^, as well as the nearby band at 1160 cm^–1^, increase. These changes indicate variations in the phosphate functionalities after adsorption. It can be attributed to increased loading of nonferrihydrite-bonded exDNA within the adsorption complex, which is consistent with the adsorption results and supports the adsorbate multilayer formation on the surface. Alternatively, the co-adsorption of phospholipids or polysaccharides present in bacterial biomass occurs,^24^ as bands from these molecules significantly overlap.^84,85^

Upon the formation of exDNA-ferrihydrite conjugates within bacterial biomass, their spectra exhibit protein-related bands. Specifically, bands at 1640 and 1660 cm^–1^, ascribed to the amide I (C=O stretching modes),^16,75,86^ increase inintensity as shoulder bands of the adsorbed water band at 1630 cm^–1^. The presence of protein in the conjugates is further indicated by the amide II band, observed at 1555 and 1509 cm^–1^, corresponding to the stretching modes of the N–H and C=O bonds in the CO–NH groups of proteins,^77^ the COO^–^ related band at 1416 cm^–1^,^77^ and the C–H_2_ bending mode distinguished at 1455 cm^–1^.^87^ Additionally, the appearance of stretching N–H overtones at 3180 and 3250 cm^–1^ confirms their presence,^88^ but they significantly overlap with stretching −OH-related modes in the 3100–3500 cm^–1^ region.^89^ The observed increase in the intensity of protein-related bands with rising bacterial biomass concentration during adsorption suggests their incorporation into the adsorption complex and involvement in the formation and development of supramolecular assemblies and ferrihydrite conjugates. This is consistent with zeta potential measurements, indicating enhanced incorporation of molecules bearing positively charged functional groups. This process also appears to occur in the absence of exDNA, as the protein bands are present in the Fr-DL sample, where no additional exDNA was introduced to the system, and ferrihydrite was exposed only to bacterial biomass (Figure 4).

In addition to proteins, the bands at 1430 and 1455 cm^–1^ associated with COO^–^ and CH_2_, respectively, may also be ascribed to phospholipids.^84^ Their presence is further indicated by the low-intensity 1730 cm^–1^ band associated with COOH stretching,^90^ previously determined phosphate-related groups, whose intensity increases with bacterial biomass concentration in the system, as well as the 2850 and 2918 cm^–1^ bands, associated with C–H vibrations of CH_3_ and CH_2_ functional groups.^87,91^ Thus, phospholipids may be incorporated into conjugates.

The spectral characteristics of ferrihydrite after exDNA adsorption in bacterial biomass indicate co-adsorption and incorporation into the supramolecular associations of proteins and possibly phospholipids and polysaccharides. In general, the intensity of biomolecule-related bands increases relative to the background signal with an increasing bacterial biomass concentration, supporting the hypothesis that an increasing amount of biomolecules is incorporated into the adsorption complex. For comparison, the FTIR spectra of raw exDNA and bacterial biomass are shown in Figure S11.

#### 3.3.2 AFM and s-SNOM Analysis

Advanced spectroscopic imaging using s-SNOM enabled us to gain additional insights into the spatial distribution of specific spectral bands within ferrihydrite conjugates upon biomolecule adsorption. The AFM and s-SNOM images of the Fr, Fr-DNA, Fr-DNA-ML, and Fr-DNA-DL samples, with a unified signal scale are shown in Figure 5. For each material, imaging was conducted using bands representing functionalities characteristic of specific biomolecules, which include 1060 cm^–1^ (phosphate/C–O or C–C —exDNA, phospholipids, and polysaccharides), 1450 cm^–1^ (C–H_2_—phospholipids and proteins), 1555 cm^–1^ (amide II—proteins), and 1620–1660 cm^–1^ (amide I—proteins or adsorbed water) bands. It should be emphasized that in the nanoscale s-SNOM measurements, signal intensity depends on both the local concentration of the target moiety and the thickness of the analyzed object. Thus, it is related to spectral characteristics and the height of the analyzed object.

**Figure 5:**
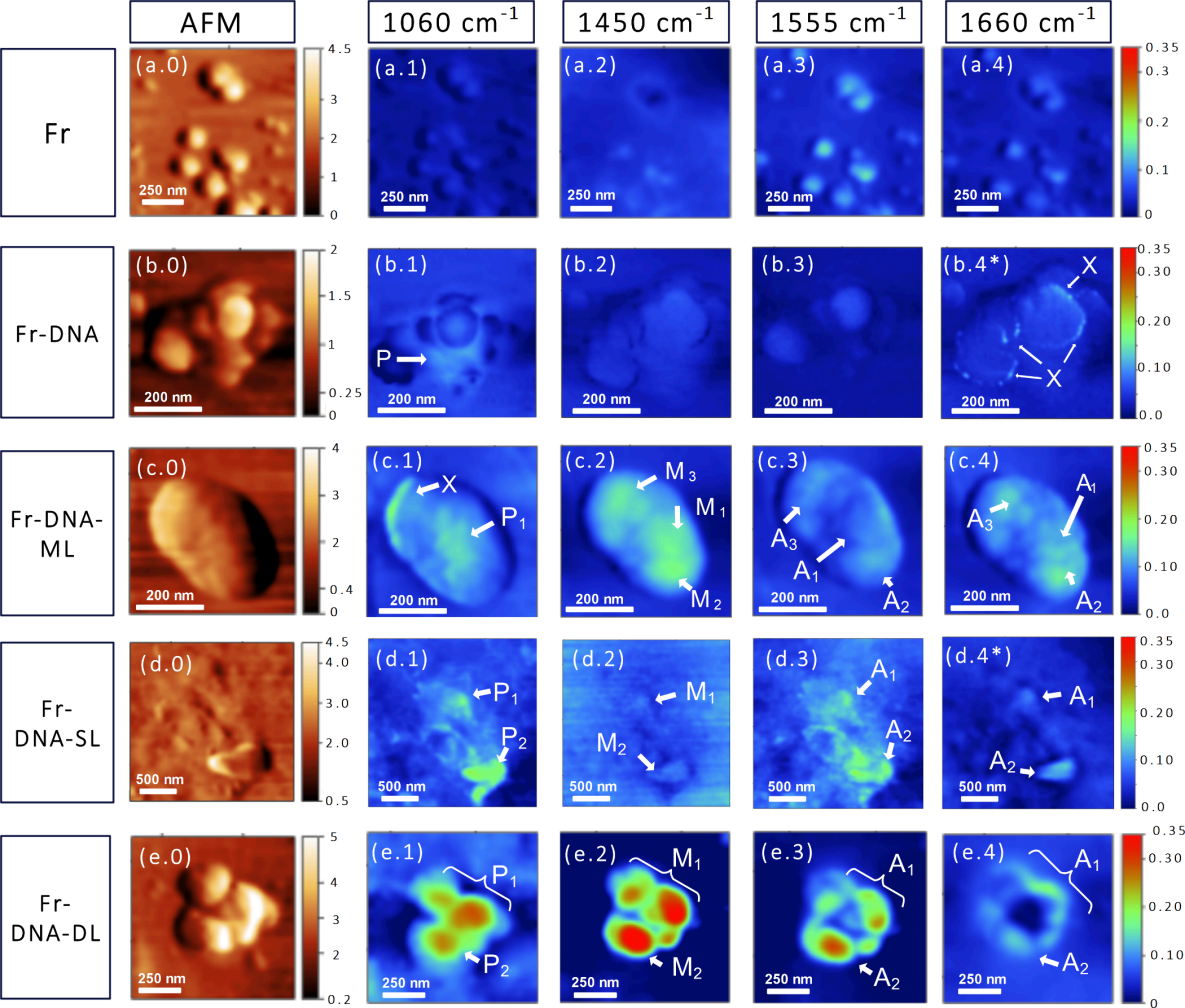
AFM and s-SNOM images of Fr (a.0–5), Fr-DNA (b.0–5), Fr-DNA-ML (c.0–5), Fr-DNA-SL (d.0–5), Fr-DNA-DL (e.0–5) mapped at 1060, 1450, 1555, and 1620 cm^–1^. (P—phosphate/C–O or C–C bands accumulation spots, M—methylene band accumulation spots, A—amide I or amide II bands accumulation spots, X—analytical artifact resulting from the high amplitude of sample height). *Map represents the 1630 cm^–1^ band.

AFM shows ferrihydrite in the form of aggregates (Figure 5a.0), which agrees with TEM images (Figure S5a) and s-SNOM images indicate a relatively uniform signal intensity distribution of the examined bands. The highest intensity was determined for 1555 and 1660 cm^–1^ bands (Figure 5 a.3–4), which correspond to the FTIR results (Fr—Figure 4) and are related to carbonate and adsorbed water.^92^ Fr-DNA is visualized as a conjugate of multiple smaller aggregates by AFM (Figure 5 b.0), and its spectral mapping delivers interesting results. A 1060 cm^–1^ band signal increase is noted within the areas between particular ferrihydrite aggregates (Figure 5b.1—P area), while signals for other bands are markedly lower (Figure 5b.2–4). The pronounced signal at 1060 cm^–1^ may suggest accumulation of exDNA in determined areas, as it is a characteristic band for exDNA (Figure 4), and no other biomolecules are present in the system. The formation of larger conjugates and the accumulation of exDNA between individual ferrihydrite aggregates underscore the role of exDNA as a bridging agent during the conjugate formation process.^14,47^

The morphological and spectral characteristics of ferrihydrite conjugates change when they are formed in a system simultaneously containing exDNA and bacterial biomass. The Fr-DNA-ML sample is represented by an aggregate with a smooth surface and no distinguishable smaller aggregates (Figure 5c.0). The 1060 cm^–1^ band signal seems to concentrate in the middle of the conjugate (P_1_ area), along with a signal form CH_2_ band at 1450 cm^–1^ (M_1_ area) and weaker signals at 1555 and 1620 cm^–1^ (A_1_ area) (Figure 5c.1–4). Additionally, all 1455, 1555, and 1660 cm^–1^ bands appear to accumulate in two more regions, A_2_/M_2_ and A_3_/M_3_, located at the upper and lower ends of the conjugate, with a lower contribution from the 1060 cm^–1^ band. This indicates the potential spatial differentiation of biomolecules distribution in the conjugate. As this sample represents conjugates formed at a relatively low bacterial biomass concentration (Fr-DNA-ML), the band at 1060 cm^–1^ most likely corresponds to an exDNA-enriched region (P_1_). Thus, the upper and lower parts of conjugates may be depleted in exDNA and contain a greater amount of molecules from the bacterial biomass matrix, e.g., proteins or phospholipids. These combined results indicate that co-adsorption of all types of biomolecules occurs even at low bacterial biomass loading (as the 1455, 1555, and 1660 cm^–1^ bands are absent in the Fr-DNA sample). Moreover, they appear to occupy different regions of the conjugates to some extent, underscoring the direct interaction between the ferrihydrite surface and the biomolecules.

The Fr-DNA-SL sample, representing conjugates formed at a moderate bacterial biomass concentration, exhibits similar spectral characteristics to the Fr-DNA-ML sample, with only a lower signal at the 1450 cm^–1^ band. However, all bands appear to exhibit greater spatial homogeneity (especially at 1060 and 1555 cm^–1^) compared to the Fr-DNA-ML sample. Enhanced co-occurrence of the examined bands may indicate the greater loading of biomolecules into conjugates and a higher degree of co-localization of biomolecules derived from bacterial biomass and exDNA within the same regions of the conjugate, compared to the Fr-DNA-ML sample. Possibly, co-adsorption of polysaccharides and phospholipids also occurs, increasing the signal of the 1060 cm^–1^ band. However, acknowledging that Fr-DNA-SL represents a sample in which the zeta potential of conjugates in the system begins to rise (Figure 2), and exDNA adsorption capacity onto ferrihydrite is enhanced, the co-localization of amide- and phosphate-related bands supports the hypothesis that proteins may act as a facilitating agent for exDNA immobilization within conjugates.

Ferrihydrite upon the adsorption of exDNA at the maximal tested bacterial biomass loading, represented by the Fr-DNA-DL sample (Figure 5e.0–4), shows a significantly higher signal from 1060, 1450, and 1555 cm^–1^ bands and the highest degree of spatial band homogeneity among examined samples. The obtained results indicate that two processes occur simultaneously. First, the formation of biomolecular assemblies and their adsorption onto ferrihydrite are intensified compared to Fr-DNA-ML and Fr-DNA-SL. Second, the incorporated biomolecules in conjugates may exhibit overlapping characteristic bands, thereby resulting in signal enhancement. Both processes suggest that, with increasing bacterial biomass concentration in the system, the amount of biomolecules incorporated into conjugates, as well as their co-location, increases. This supports the narration in Figure 2d, which states that all types of biomolecules are incorporated into ferrihydrite conjugates and that exDNA immobilization is enhanced with an increasing biomolecule concentration.

## 4 Conclusions

The outcomes of this study offer valuable insights into the key mechanisms driving the adsorption of exDNA onto ferrihydrite in the presence of bacterial biomolecules. We studied how lysed bacterial biomass influences the formation of biomolecule-ferrihydrite conjugates and how this process affects the immobilization of exDNA. Generally, as the bacterial biomass concentration increases, exDNA adsorption onto ferrihydrite is facilitated. By studying the ζ-potential of the formed conjugates, three phases of the process are distinguished. In Phase I, where the bacterial biomass concentration is relatively low, exDNA immobilization on the ferrihydrite surface is accompanied by the co-adsorption of biomolecules from bacterial biomass onto ferrihydrite, thereby decreasing the ζ-potential of the particles in the system. At low bacterial biomass loading, s-SNOM images show slight spatial variations in spectral intensity between the exDNA and other biomolecule-related bands, suggesting that, to some extent, the exDNA and biomolecules tend to occupy different regions of the conjugates. When the bacterial biomass concentration increases, Phase II begins, during which positively charged proteins facilitate the formation of supramolecular assemblies and the immobilization of exDNA within ferrihydrite conjugates, as evidenced by the rising ζ-potential of the conjugates and the greater spectra homogeneity of examined bands in s-SNOM images. As the bacterial biomass continues to increase, the system enters Phase III, during which the growth propagation of biomolecular assemblies becomes more pronounced. This results in increased protein incorporation into conjugates and the formation of numerous positively charged adsorption-active centers, thereby further significantly enhancing the immobilization of exDNA.

During bacterial cell lysis, a variety of biomolecules are released into the surrounding microenvironment. Upon release from cells, these biomolecules may react with other compartments of organic matter and also with mineral species. A biomolecule with a critical role in environmental processes is exDNA, which carries and stores genetic information. Therefore, processes involving interactions of exDNA with organic matter and minerals may govern the fate of genes in the environment by controlling exDNA stability and HGT potential. Our results indicate that ferrihydrite may be an important factor facilitating the immobilization of exDNA, as a common soil and sediment mineral.^93^ Furthermore, the presence of biomolecules, particularly proteins, tends to enhance exDNA adsorption. The determined interactions in the biomolecules–exDNA–ferrihydrite system may play crucial roles in biofilm formation, the organization of organic matter in soils and sediments, and the exchange of genes in these environments. More importantly, determined processes may govern the fate and dissemination of antibiotic resistance genes, which constitute an emerging environmental and public health issue.^94^ This study provides an initial perspective on the mechanisms governing the exDNA reactivity in environmental matrices. The key future direction in this area is to investigate the potential of exDNA to act as a vector for horizontal gene transfer, both when confined in biomolecular assemblies and when adsorbed to mineral surfaces.

## Supplementary Material



## Notes

DNA gel electrophoresis results, XRD pattern for ferrihydrite, FTIR spectra of raw ferrihydrite, N_2_ adsorption analysis results for raw ferrihydrite, zeta potential for raw ferrihydrite, methods used for raw ferrihydrite analysis, equation used for adsorption kinetics and isotherms modeling, exDNA adsorption isotherm onto ferrihydrite, sedimentation kinetics curves of ferrihydrite and ferrihydrite after adsorption with and without a bacterial biomass matrix, TEM images for ferrihydrite after adsorption of exDNA with and without bacterial biomass, the influence of bacterial biomass concentration on the adsorption capacity of exDNA on ferrihydrite, electrical conductivity in the solution samples with varying bacterial biomass concentrations, zeta potential of raw exDNA and bacteria biomass solutions, exDNA adsorption kinetics onto ferrihydrite, adsorption kinetics modeling results, the influence of pH on the exDNA adsorption with and without the presence of bacterial biomass, and FTIR spectra of exDNA and bacterial biomass (PDF)
