# Supplementary material for: Bacterial Biomolecules Drive Extracellular DNA Adsorption onto Ferrihydrite: Interfacial Interactions and Spectroscopic Insights
Source: Langmuir. 2026 Jun 2;42(23):16131–44. doi: 10.1021/acs.langmuir.6c00302 (PMC13276911; doi:10.1021/acs.langmuir.6c00302)
Supplement: Supplementary file 1 [file la6c00302_si_001.pdf]

Supporting Information

Bacterial biomolecules drive extracellular DNA adsorption onto ferrihydrite: interfacial interactions and spectroscopic insights

Mateusz Skalny[1], Jakub Czeremuga[1], Maciej Roman[2], Tomasz P. Wróbel[2], Lukasz Dziewit[3], Tomasz Bajda[1]*

[1]Faculty of Geology, Geophysics and Environmental Protection, AGH University of Krakow, Mickiewicza 30, 30-059, Krakow, Poland

[2]Laboratory of Applied Microbial Ecology, Institute of Bioengineering, Faculty of Biology, University of Warsaw, Miecznikowa 1, 02-096 Warsaw, Poland

[3]SOLARIS National Synchrotron Radiation Centre, Jagiellonian University, 98 Czerwone Maki Str., 30-392, Krakow, Poland

*** Corresponding author:**

E-mail address: mskalny@agh.edu.pl;

Table of contents

Figure S1. exDNA length distribution by gel electrophoresis. 3

Figure S2. XRD pattern (a), FTIR spectra (b), N_2 hysteresis curve (c), pore size distribution (d), zeta potential (e) for synthesized ferrihydrite. 4

Methods S1. Debye-Scherrer equation 5

Table S1. Textural characteristics of synthesized ferrihydrite. 5

Methods S2. Materials characterization 5

Methods S3. Isotherms and kinetics modeling 6

Figure S3. exDNA adsorption isotherm 7

Table S2. Results of Langmuir and Freundlich model fits to adsorption isotherm data. 7

Figure S4. Sedimentation kinetics curves of ferrihydrite and ferrihydrite after adsorption with and without bacterial biomass matrix. 8

Figure S5. TEM images of Fr, Fr-DNA, Fr-DNA-ML, Fr-DNA-SL,, and Fr-DNA-DL under two varying magnifications. 9

Figure S6. The influence of bacterial biomass concentration on the adsorption capacity of exDNA on ferrihydrite. 10

Figure S7. Electrical conductivity in the samples of solution samples with varying bacterial biomass concentrations. 10

Figure S8. Zeta potential of raw exDNA and bacteria biomass solutions. 11

Figure S9. exDNA adsorption kinetics onto ferrihydrite with and without bacterial biomass concentration of 1.04 µg/mL and 3.51 µg/mL 11

Table S3. Adsorption kinetics model of exDNA adsorption on ferrihydrite. 12

Figure S10. The influence of pH on the exDNA adsorption with and without the presence of bacterial biomass at concentrations of 1.04 and 3.51 µg/mL. 12

Figure S11. FTIR spectra of exDNA (a) and bacterial biomass (b) used in the study. 13

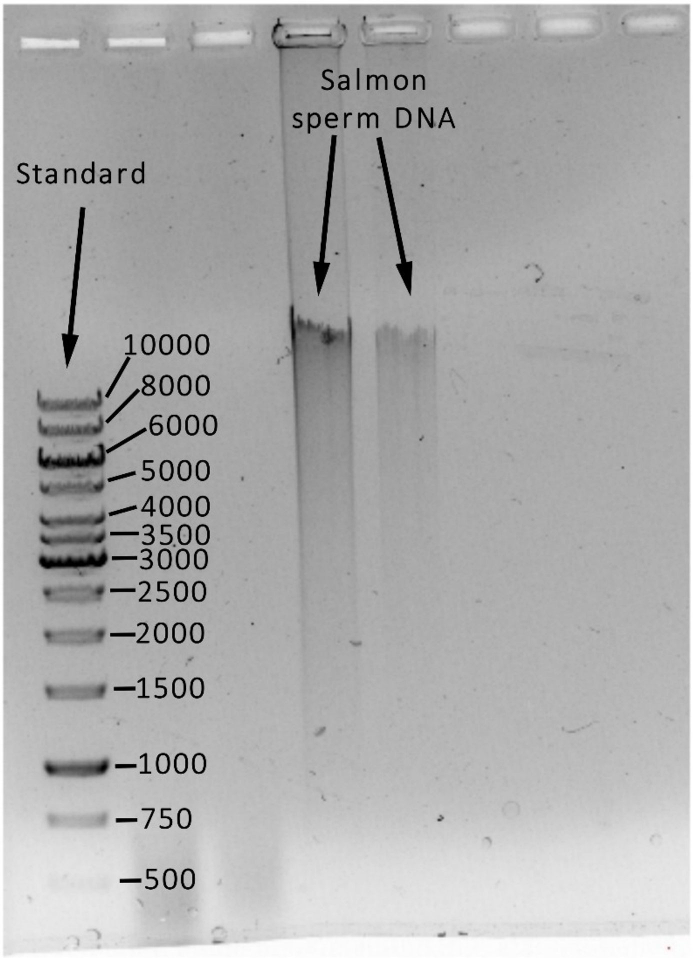

Figure S1. exDNA length distribution by gel electrophoresis.

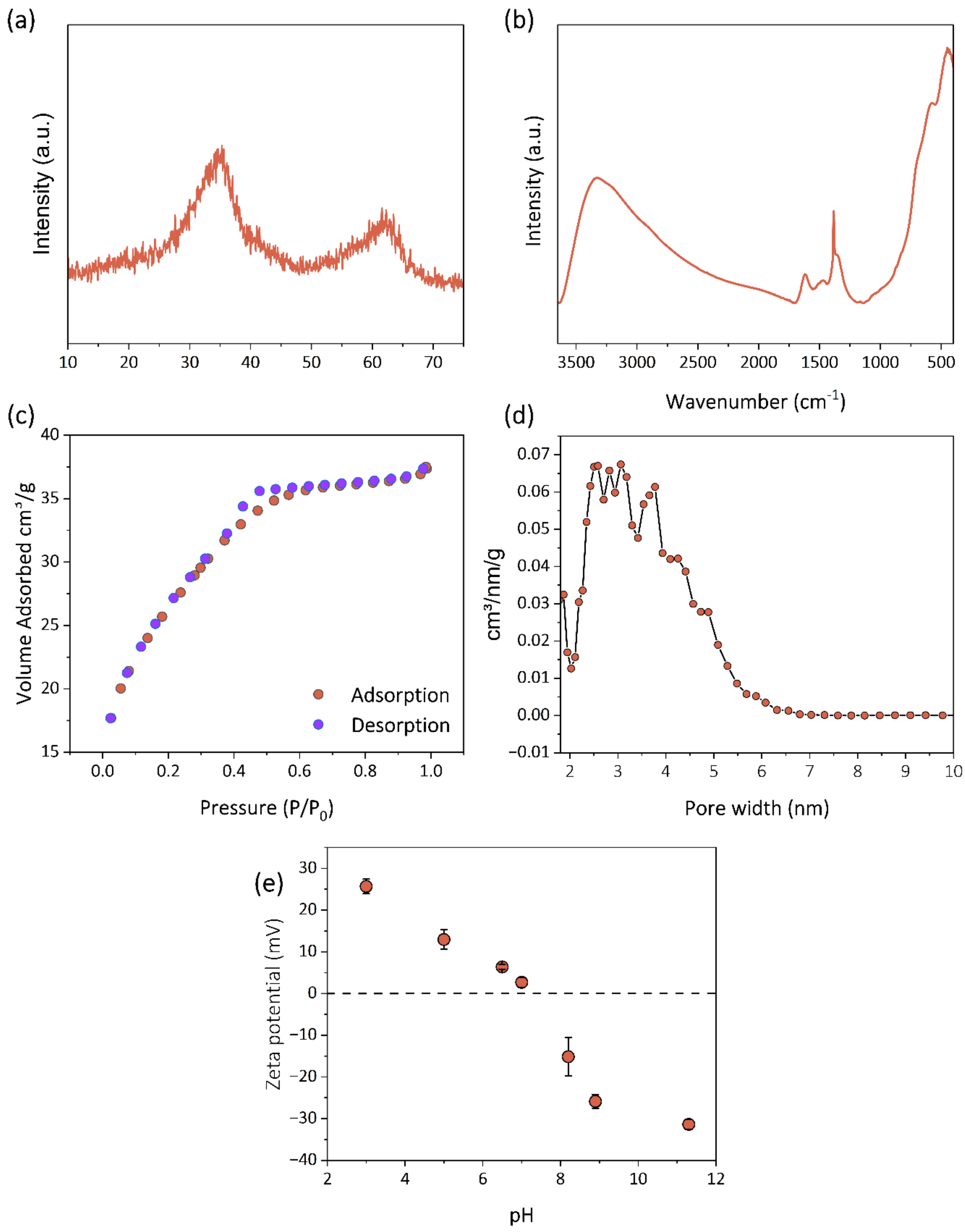

Figure S2. XRD pattern (a), FTIR spectra (b), N_2 hysteresis curve (c), pore size distribution (d), zeta potential (e) for synthesized ferrihydrite.

Methods S1. Debye-Scherrer equation

$$D = \frac{k\lambda}{\beta cos\theta} \text{ (Eq. S1)}$$

Where D is the average crystallite size (nm), λ is the X-ray wavelength (0.15406 nm for Cu Kα), β is the full width at half maximum of the intense and broad peaks, and θ is the Bragg's angle.

Table S1. Textural characteristics of synthetized ferrihydrite.

**S_{BET} [m²/g]**	**$V_{tot}^{0.99}$ [cm³/g]**	**V_{mic}^{DR} [cm³/g]**	**V_{mes}^{BJH} [cm³/g]**
316.6	0.188	0.0347	0.152

Methods S2. Materials characterization

The phase composition of ferrihydrite was analyzed by X-ray diffraction (XRD) using a SmartLab RIGAKU diffractometer (RIGAKU, Tokyo, Japan) with CuKα radiation and a graphite monochromator. The Fourier Transform Infrared Spectroscopy (FTIR) spectra were collected using a Nicolet 6700 spectrometer (Thermo Fisher, Waltham, MA, USA) in the 4000 - 400 cm^{-1} range at a resolution of 4 cm^{-1}. The specific surface area (SSA) and porosity of materials were determined using N_2 gas adsorption/desorption isotherms at 77 K using an ASAP 2020 apparatus (Micromeritics, Norcross, GA, USA). The samples were outgassed for 24 h at 373 K. The BET equation was used for the specific surface area calculations (SBET) [1]. The total pore volume ($V_{tot}^{0.99}$) was calculated from N_2 adsorbed at a relative vapor pressure (P/P_0) ~0.99. The volume of the micropores (V_{mic}^{DR})was calculated by applying the Dubinin–Radushkevich method [2]. The mesopore volume (V_{mes}^{BJH}) was determined from the adsorption branch of the isotherms by using the BJH (Barrett–Joyner–Halenda) method [3] in the mesopore range proposed by Dubinin [2]. The macropore volume (V_{mac}) was calculated using the Eq S2:

$$V_{mac} = V_{tot}^{0.99} - (V_{mic}^{DR} + V_{mes}^{BJH}) \text{ (Eq. S2)}$$

V_{mic}^{DR} – the volume fo micropores

V_{mes}^{BJH} – the volume of mesopores

Methods S3. Isotherms and kinetics modeling

The experimentally obtained data from adsorption kinetics were modeled using the Pseudo-first order and Pseudo-second order equations:

Langmuir model

$$\frac{C_{eq}}{q_{eq}} = \frac{C_{eq}}{q_{max}} + \frac{1}{K_L * q_{max}} \text{ (Eq. S2)}$$

q_{eq} – amount adsorbed by zeolite at equilibrium [mg/g]

C_{eq} – the equilibrium concentration in solution, [mg/L]

q_{max} – monolayer capacity of the adsorbent, [mg/g]

K_L – the Langmuir adsorption constant, [L/mg]

Freundlich model

$$\ln(q_{eq}) = \ln(K_F) + \frac{1}{n}\ln(C_{eq}) \text{ (Eq. S3)}$$

q_{eq} – amount adsorbed by carbon at equilibrium [mg/g]

C_{eq} – the equilibrium concentration in solution [mg/L]

K_F – the Freundlich constant [$mg^{1-1/n}\,L^{1/n}$ /g]

1/n – heterogeneity factor [-]

Pseudo-first order model

$$\ln(q_{eq} - q_t) = \ln(q_{eq}) - k_1 t \text{ (Eq. S4)}$$

t – time [min]

q_t – substance amount adsorbed at time *t* [mg/g]

q_{eq} – substance amount adsorbed derived from equation [mg/g]

k_1 – model constant of adsorption [min^{-1}]

Pseudo-second order model

$$\frac{t}{q_t} = \frac{1}{k_2 * q_{eq}^2} + \frac{t}{q_{eq}} \text{ (Eq. S5)}$$

t – time [min]

q_t – substance amount adsorbed at time t [mg g^{-1}]

q_{eq} – substance amount adsorbed at equilibrium [mg g^{-1}]

k_2 – model constant of adsorption [g mg^{-1} min^{-1}]

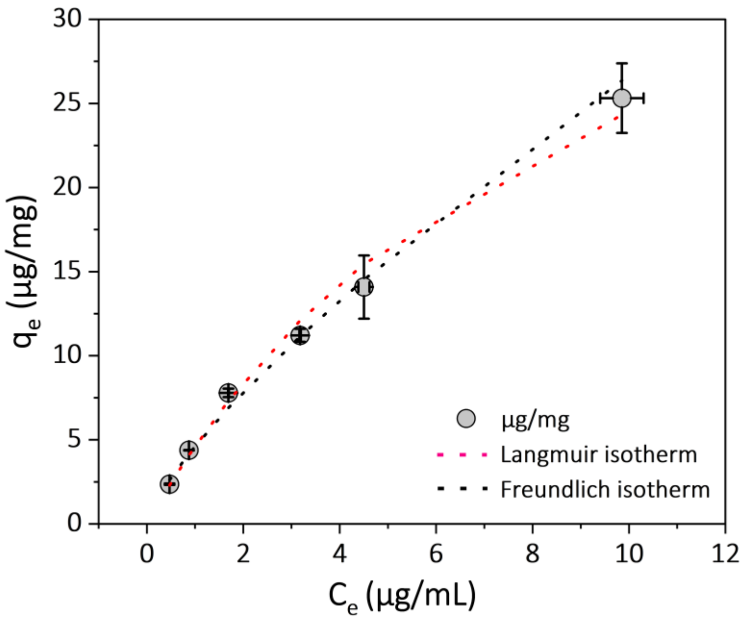

Figure S3. exDNA adsorption isotherm (ferrihydrite dispersion concentration: 0.2 mg/mL, reaction time: 60 min, exDNA C_0: 0.5–10 µg/mL)

Table S2. Results of Langmuir and Freundlich model fits to adsorption isotherm data.

Langmuir model			***Freundlich model***		
**Q_{max} [µg/mg]**	**K_L [ml/mg]**	**R^2**	**N**	**Kf (mg/g)/(ml/mg)**	**R^2**
46.99	0.10	0.93	1.31	4.6	0.99

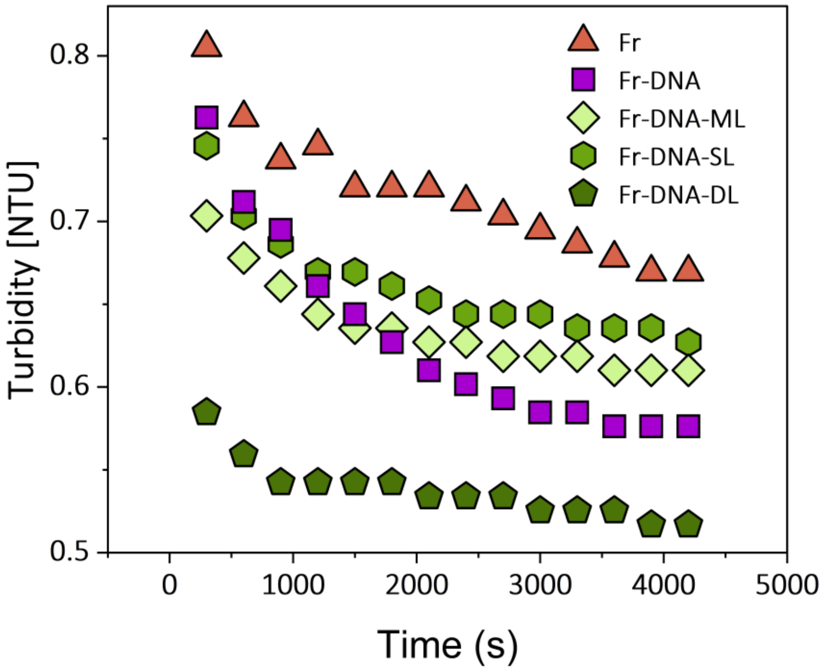

Figure S4. Sedimentation kinetics curves of ferrihydrite and ferrihydrite after adsorption with and without bacterial biomass matrix. Fr - raw ferrihydrite, Fr-DNA - ferrihydrite after exDNA adsorption, Fr-DNA-ML - ferrihydrite after exDNA adsorption in bacterial biomass medium with concentration of 1.04 µg/mL, Fr-DNA-SL ferrihydrite after exDNA adsorption in bacterial biomass medium with concentration of 3.51 µg/mL, Fr-DNA-DL - ferrihydrite after exDNA adsorption in bacterial biomass medium with concentration of 14.03 µg/mL.

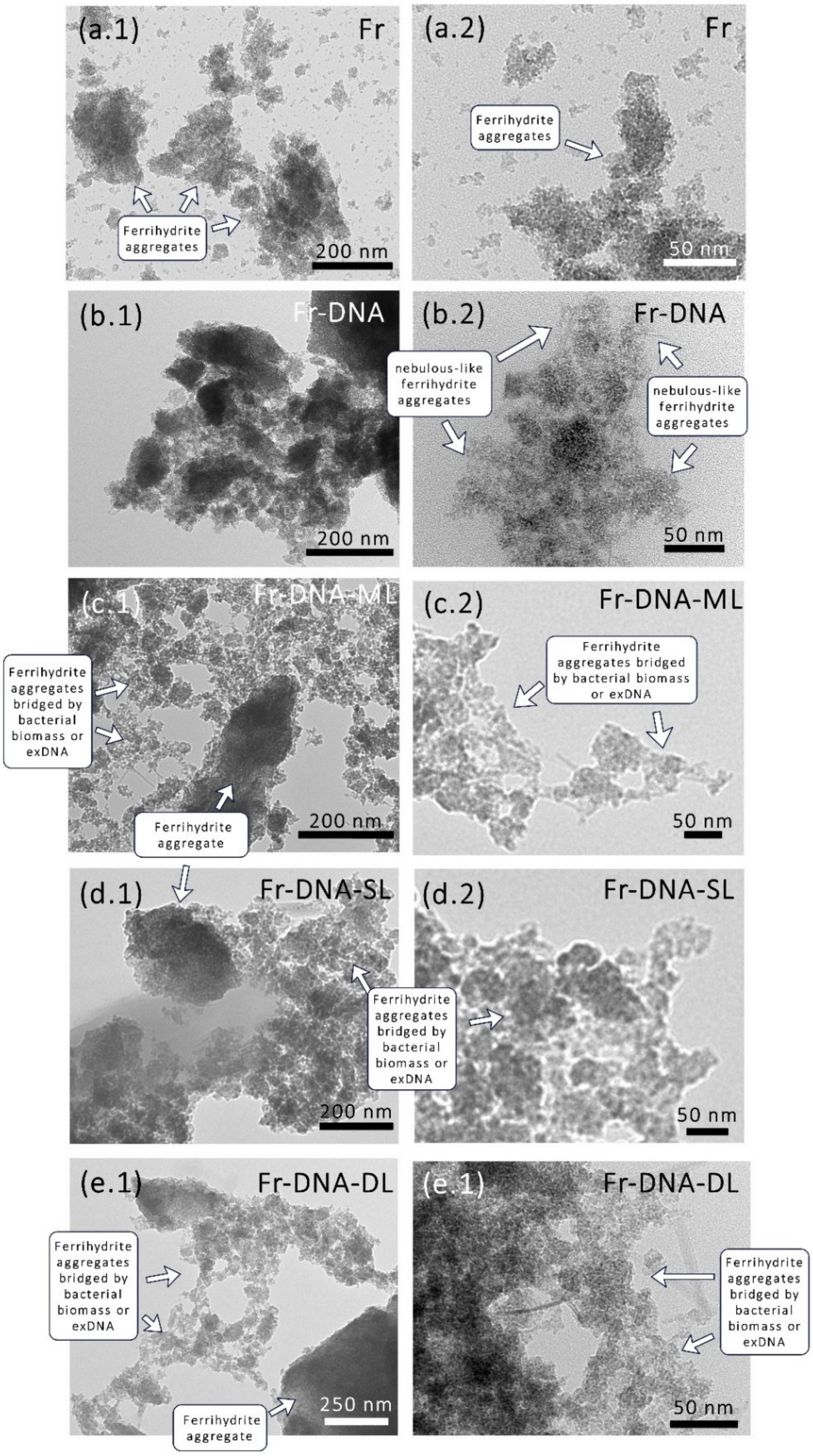

Figure S5. TEM images of Fr (a.0, b.0), Fr-DNA (a.1, b.1), Fr-DNA-ML (a.2, b.2), Fr-DNA-SL (a.3, b.3), and Fr-DNA-DL (a.4, b.4) under two varying magnifications.

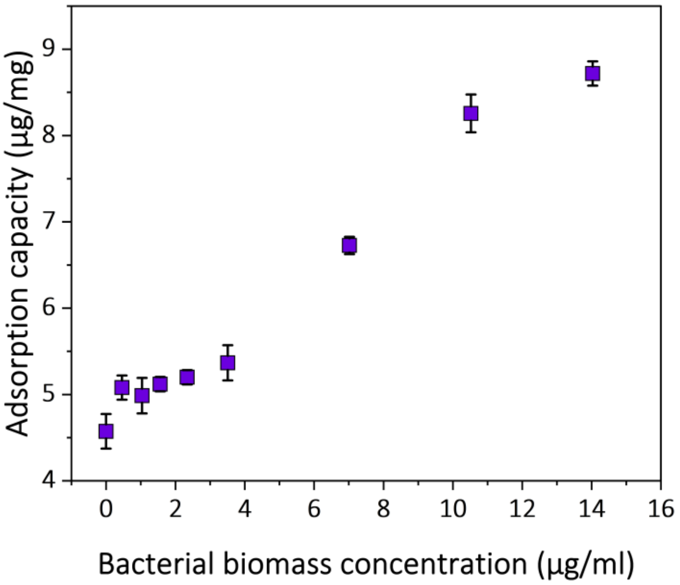

Figure S6. The influence of bacterial biomass concentration on the adsorption capacity (mg/g) of exDNA on ferrihydrite.

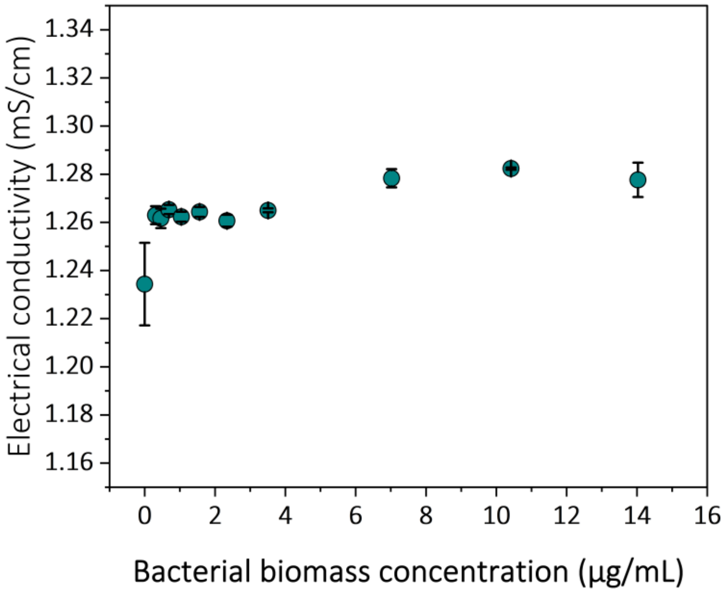

Figure S7. Electrical conductivity in the solution samples with varying bacterial biomass concentrations.

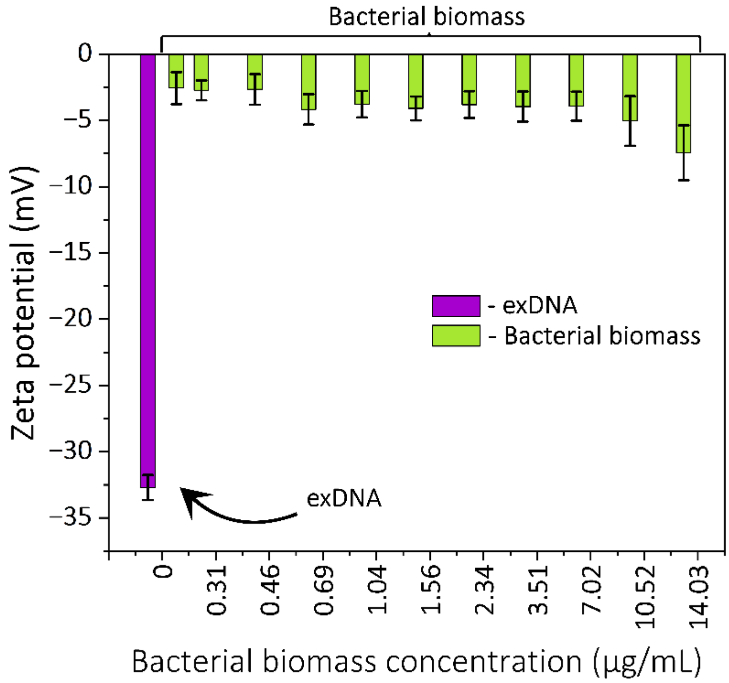

Figure S8. Zeta potential of raw exDNA and bacteria biomass solutions.

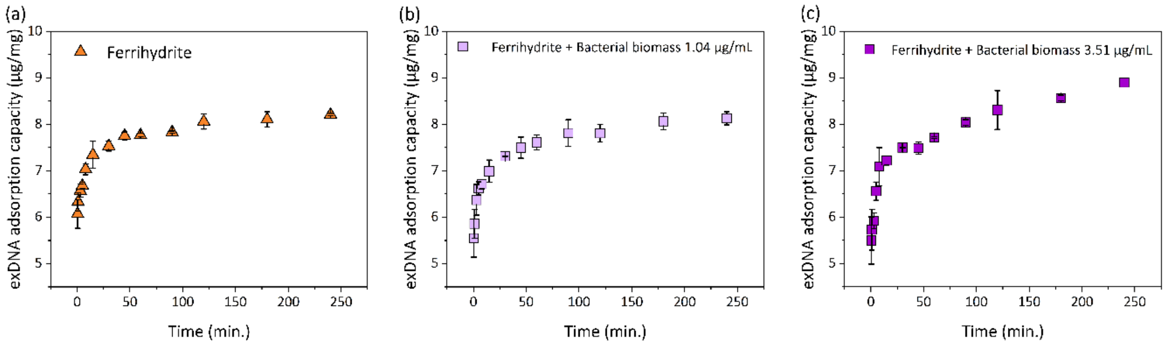

Figure S9. exDNA adsorption kinetics onto ferrihydrite (a) with and without bacterial biomass concentration of 1.04 μg/mL (b) and 3.51 μg/mL (c). Error bars represent the standard deviation of independent samples.

Table S3. Adsorption kinetics model of exDNA adsorption on ferrihydrite.

Adsorbate	Bacterial biomass concentration (μg/mL)	Pseudo-first order			Pseudo-second order		
		q_e *(mg/g)*	k_1	R^2	q_e *(mg/g)*	k_2	R^2
exDNA	0	7.50 ± 0.17	2.870 ± 0.60	0.924	7.66 ± 0.13	0.715 ± 0.18	0.958
	1.04	7.33 ± 0.18	2.347 ± 0.48	0.912	7.52 ± 0.14	0.541 ± 0.13	0.953
	3.51	7.54 ± 0.25	2.047 ± 0.53	0.850	7.81 ± 0.2	0.387 ± 0.11	0.912

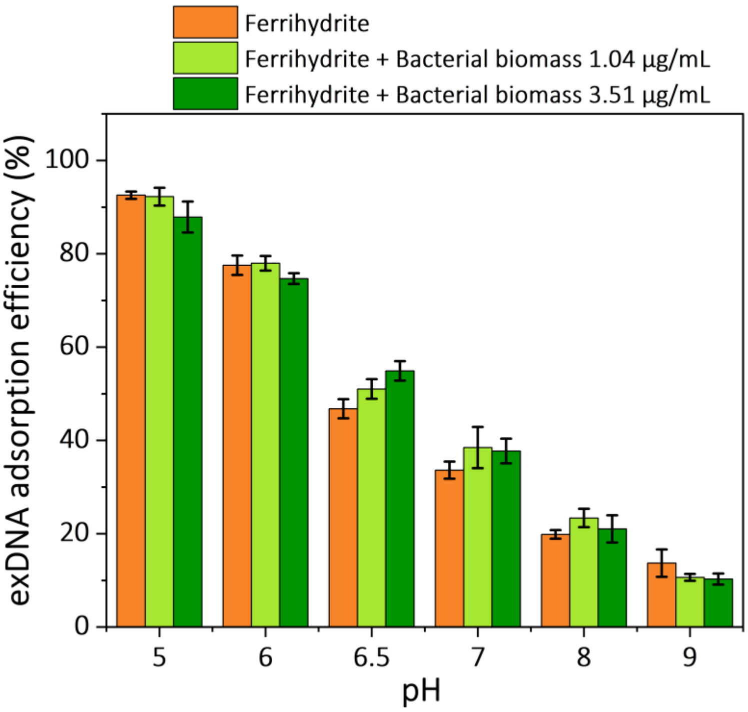

Figure S10. The influence of pH on the exDNA adsorption with and without the presence of bacterial biomass at concentrations of 1.04 and 3.51 μg/mL (exDNA C_0: 0.5 μg/mL, reaction time: 60 min, ferrihydrite concentration: 0.1 mg/mL).

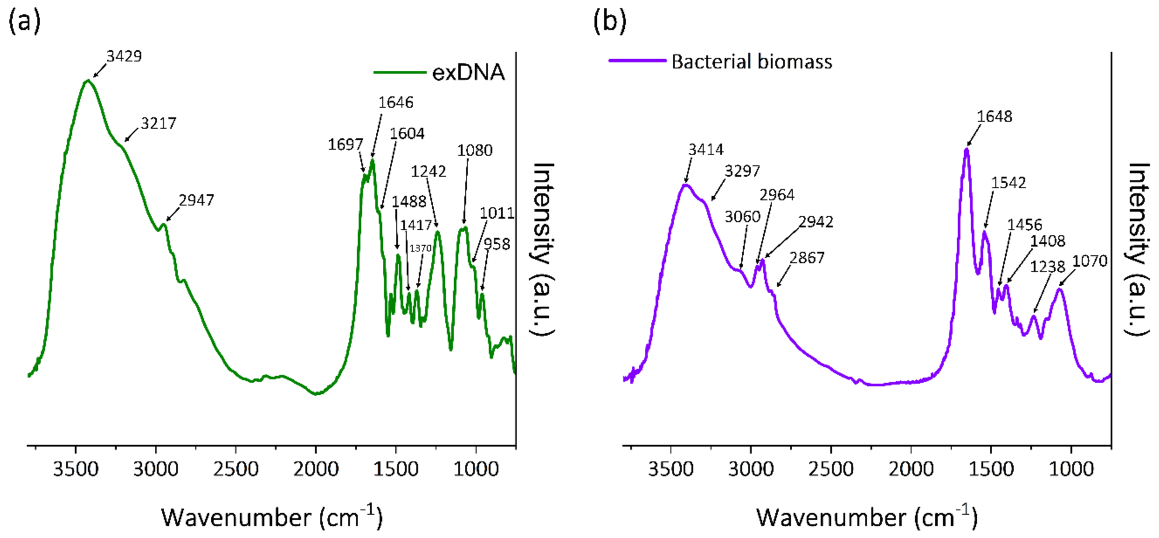

Figure S11. FTIR spectra of exDNA (a) and bacterial biomass (b) used in the study.

References

[1] S. Brunauer, P. H. Emmett, and E. Teller, "Adsorption of Gases in Multimolecular Layers," *J Am Chem Soc*, vol. 60, no. 2, pp. 309–319, Feb. 1938, doi: 10.1021/ja01269a023.

[2] M. M. Dubinin, "The Potential Theory of Adsorption of Gases and Vapors for Adsorbents with Energetically Nonuniform Surfaces.," *Chem Rev*, vol. 60, no. 2, pp. 235–241, Apr. 1960, doi: 10.1021/cr60204a006.

[3] E. P. Barrett, L. G. Joyner, and P. P. Halenda, "The Determination of Pore Volume and Area Distributions in Porous Substances. I. Computations from Nitrogen Isotherms," *J Am Chem Soc*, vol. 73, no. 1, pp. 373–380, Jan. 1951, doi: 10.1021/ja01145a126.
